# Effects of Nano-Hydroxyapatite-Coated PRF on Gingiva-Derived Mesenchymal Stem Cells: In Vitro Study

**DOI:** 10.3390/ijms27135736

**Published:** 2026-06-25

**Authors:** İzzet Melih Gürkan, Bahar Demir Cevizlidere, Seçil Çalişkan, Sibel Özdemir, Hakan Özdemir

**Affiliations:** 1 Department of Periodontology, Faculty of Dentistry, Eskişehir Osmangazi University, Eskişehir 26140, Türkiye; hozdemir52@hotmail.com; 2 Department of Stem Cell, Cellular Therapy and Stem Cell Production Application and Research Centre, Eskisehir Osmangazi University, Eskişehir 26140, Türkiye; bahardemircevizlidere@gmail.com; 3 Department of Pedodontics, Faculty of Dentistry, Eskişehir Osmangazi University, Eskişehir 26140, Türkiye; secilcaliskan@ogu.edu.tr; 4 Department of Neurology, Yunus Emre State Hospital, Eskişehir 26140, Türkiye; drsibelkaraca@hotmail.com

**Keywords:** platelet-rich fibrin, nano-hydroxyapatite, gingiva-derived mesenchymal stem cells, regenerative dentistry, tissue engineering

## Abstract

Platelet-rich fibrin (PRF) has been widely used in regenerative dentistry because of its potential to support tissue regeneration. Recently, modifications in PRF preparation protocols and tube surface characteristics have attracted attention because of their possible influence on fibrin organization and biologic activity. The present in vitro study aimed to evaluate the effects of nano-hydroxyapatite platelet-rich fibrin (HA-PRF) on gingiva-derived mesenchymal stem cells (GMSCs) by comparing it with leukocyte platelet-rich fibrin (L-PRF) and titanium platelet-rich fibrin (T-PRF). Gingival tissue and venous blood samples were obtained from a systemically healthy male volunteer. PRF membranes were prepared using conventional glass tubes, nano-hydroxyapatite-coated tubes, and titanium tubes. GMSCs were isolated, characterized, and cultured with PRF membranes. Cell viability and metabolic activity were evaluated using MTT analysis. Apoptosis and necrosis rates were assessed by Annexin V/PI flow cytometry. VEGF and TGF-β1 release levels were determined by ELISA, whereas IL-1β, IL-6, and TNF-α gene expression levels were analyzed using qRT-PCR. The HA-PRF and L-PRF groups demonstrated higher cell viability values compared with the T-PRF group on day 7. Annexin V/PI analysis revealed no statistically significant differences between the groups in terms of apoptosis and necrosis. Growth factor release and cytokine gene expression profiles demonstrated time-dependent biologic responses in all PRF membranes. Within the limitations of this study, HA-PRF showed no evidence of cytotoxicity and demonstrated biologic responses comparable to those observed with conventional L-PRF. Both HA-PRF and L-PRF generally exhibited more favorable cellular responses than T-PRF under the present experimental conditions.

## 1. Introduction

To accelerate soft and hard tissue healing, various biomaterials have been used for many years. Among these biologic products, referred to as autologous biomaterials, platelet-rich fibrin (PRF), a second-generation platelet concentrate, plays an important role in the regulation of inflammation and promotion of wound healing through the platelets, leukocytes, and growth factors contained within its structure [1,2].

Although numerous studies have demonstrated favorable clinical outcomes associated with PRF applications, several limitations related to PRF preparation methods and tube materials have recently been emphasized [3,4,5]. During PRF formation, platelets interact with and adhere to the tube surface, and platelet activation may be significantly influenced by the physical and chemical properties of the tube material [6,7]. Therefore, the surface characteristics of blood collection tubes may affect platelet adhesion, activation, and fibrin matrix organization.

In the literature, glass tubes, silica-coated plastic tubes, titanium tubes, and additive-free plastic tubes are commonly used for PRF preparation [8,9]. Previous studies have demonstrated that tube type may influence fibrin matrix organization, platelet distribution, growth factor release, and biologic compatibility [10,11]. In particular, PRF preparations obtained from silica-coated plastic tubes may contain silica microparticles detached from the tube surface, potentially affecting fibrin network formation and inducing cytotoxic effects on surrounding cells [12,13]. These findings highlight the importance of tube material biocompatibility and surface properties during PRF preparation.

Based on these considerations, several approaches have been proposed to improve the biologic characteristics of PRF through modification of tube surfaces. PRF obtained using titanium tubes has been reported to exhibit a denser and more organized fibrin network with potentially enhanced biologic properties [9,14].

In recent years, nano-scale surface modifications have emerged as a promising strategy capable of improving cellular adhesion, proliferation, and cellular responses. Hydroxyapatite is a highly biocompatible and osteoconductive bioceramic widely used in tissue engineering applications because of its favorable effects on cellular attachment and differentiation [15,16,17]. In addition to these properties, nano-hydroxyapatite coatings may provide a larger reactive surface area that facilitates interactions between blood components and the tube surface during PRF preparation. Such interactions may promote platelet adhesion and activation, potentially influencing fibrin network formation and the subsequent release of growth factors entrapped within the PRF matrix. Therefore, coating the inner surface of glass tubes with nano-hydroxyapatite may reduce potential silica contamination associated with conventional glass tubes while simultaneously enhancing platelet activation and fibrin organization. In addition, such an approach may represent a more cost-effective alternative to titanium tubes used for T-PRF preparation.

Beyond their clinical effects on wound healing, interactions between PRF biomaterials and mesenchymal stem cells have become an important area of investigation. Previous in vitro studies have demonstrated that PRF may support cell viability, proliferation, migration, and differentiation of various stem cell populations [15,16,17]. However, most available studies have evaluated PRF preparations derived from a single tube system, whereas the biologic effects of PRF membranes obtained from surface-modified alternative tube materials on gingiva-derived mesenchymal stem cells (GMSCs) remain poorly understood.

GMSCs are considered an important cellular source in periodontal and peri-implant tissue regeneration because they can be obtained relatively easily, exhibit high proliferative capacity, and possess immunomodulatory properties [18,19,20,21]. Therefore, understanding the interaction between PRF biomaterials and GMSCs may contribute to the development of regenerative treatment strategies.

The aim of the present study was to comparatively evaluate the effects of PRF membranes obtained using different tube materials and surface properties—L-PRF (glass tube), HA-PRF (nano-hydroxyapatite-coated tube), and T-PRF (titanium tube)—on biologic parameters including cell viability, apoptosis/necrosis, growth factor release, and proinflammatory gene expression in GMSCs under in vitro conditions.

In the present study, PRF membranes were prepared using three different tube systems (L-PRF, HA-PRF, and T-PRF) and subsequently evaluated for their biologic effects on gingiva-derived mesenchymal stem cells (GMSCs). Following isolation and characterization of GMSCs, cell viability and metabolic activity, apoptosis/necrosis, growth factor release, and inflammatory cytokine gene expression were assessed on days 1, 3, and 7 to compare the biologic responses induced by the different PRF preparation systems.

## 2. Results

### 2.1. Isolation and Characterization of GMSCs

**Figure 1:**
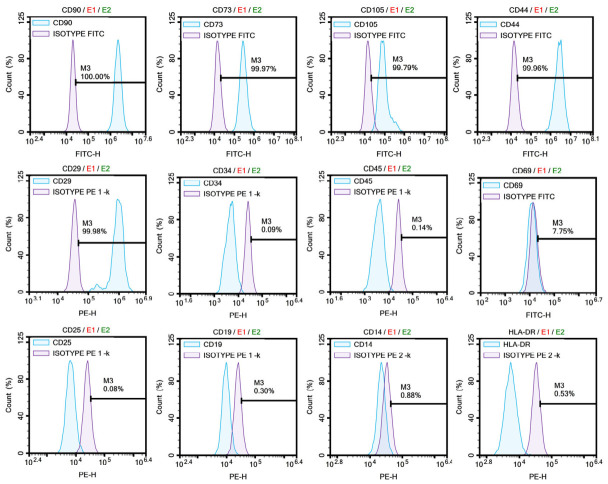
Positive expression of CD90, CD73, CD105, CD44, and CD29 and negative expression of CD34, CD45, CD69, CD25, CD19, CD14, and HLA-DR in GMSCs. In the histogram analysis, target antibodies are shown in blue, whereas isotype antibodies (negative controls) are shown in purple.

Flow cytometric analysis demonstrated that GMSCs negatively expressed hematopoietic and immunologic markers, including CD34 (0.09%), CD45 (0.14%), CD69 (7.75%), CD25 (0.08%), CD19 (0.30%), CD14 (0.88%), and HLA-DR (0.53%). In contrast, strong positive expression was observed for mesenchymal stem cell markers CD90 (100%), CD73 (99.97%), CD105 (99.79%), CD44 (99.96%), and CD29 (99.98%) (Figure 1).

**Figure 2:**
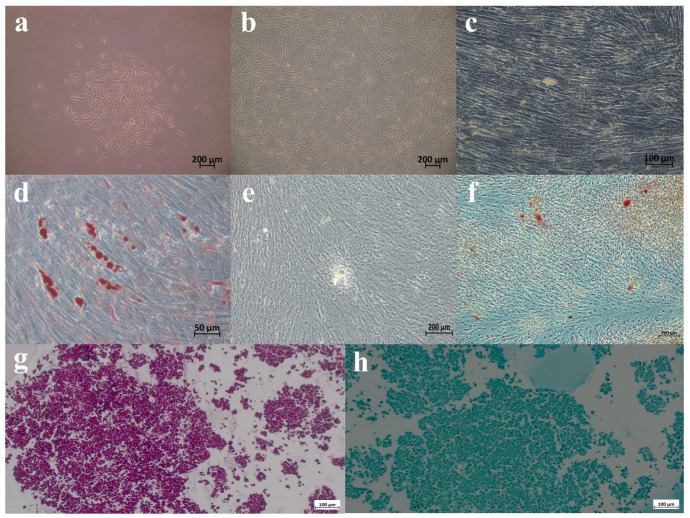
(**a**) Colony formation of adherent cells on day 7 after isolation. (**b**) Spindle-shaped, fibroblast-like morphology of isolated GMSCs. (**c**) Phase-contrast microscopic appearance of GMSCs differentiated into the adipogenic lineage on day 15. (**d**) Demonstration of intracellular neutral lipid vacuole accumulation in adipogenically differentiated cells using Oil Red O staining. (**e**) Phase-contrast microscopic appearance of GMSCs differentiated into the osteogenic lineage on day 25. (**f**) Demonstration of calcium nodule formation in osteogenically differentiated cells using Alizarin Red S staining. (**g**) Demonstration of chondrogenic tissue in chondrogenically differentiated cells using Alcian Blue staining. (**h**) Demonstration of chondrogenic cells using Safranin O staining.

Daily microscopic evaluation demonstrated that the isolated cells exhibited mesenchymal stem cell morphology. Morphologic examination revealed spindle-shaped, fibroblast-like adherent cells characteristic of gingiva-derived mesenchymal stem cells. Adipogenic differentiation was confirmed by positive Oil Red O staining and adiponectin immunocytochemical staining. Osteogenic differentiation was demonstrated by positive Alizarin Red S staining and osteocalcin immunocytochemical staining, whereas chondrogenic differentiation was confirmed by positive Alcian blue and Safranin O staining (Figure 2).

### 2.2. In Vitro Cell Viability and Metabolic Activity (MTT) Analysis

**Figure 3:**
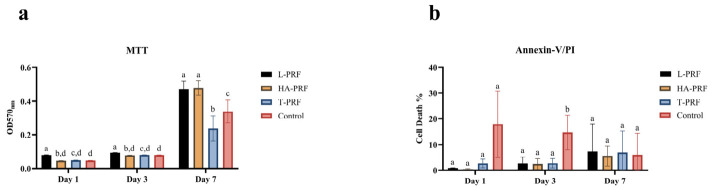
Effects of L-PRF, HA-PRF, and T-PRF membranes on GMSC viability and apoptosis at days 1, 3, and 7. (**a**) MTT assay results showing cellular metabolic activity (OD570 nm) in the experimental groups. (**b**) Annexin V/PI flow cytometric analysis showing the percentage of apoptotic and dead cells in the experimental groups. Different letters indicate statistically significant differences among groups at the same time point (*p* < 0.05).

Cell viability analysis demonstrated statistically significant differences between the groups on days 1, 3, and 7 according to membrane type (*p* < 0.05). Although statistically significant differences were detected on days 1 and 3, the magnitude of these differences was limited. More pronounced differences were observed on day 7, when both HA-PRF and L-PRF groups demonstrated higher viability values than the remaining groups; however, no statistically significant difference was observed between these two groups (*p* > 0.05) (Figure 3a).

### 2.3. Cell Death Analysis by Annexin V/PI

Cell death rates were evaluated according to the study groups. A statistically significant difference was observed among the groups on day 3 (*p* = 0.01), whereas no significant differences were detected on days 1 and 7 (*p* = 0.228 and *p* = 0.993, respectively). Post hoc analysis demonstrated higher cell death percentages in the control group than in the PRF-treated groups on day 3; however, considerable variability was observed among samples (Figure 3b).

### 2.4. Evaluation of VEGF and TGF-β1 Release

**Figure 4:**
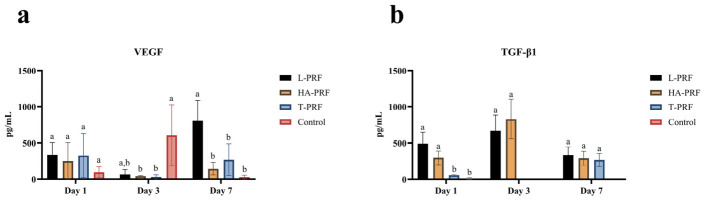
Growth factor release levels in the control, L-PRF, HA-PRF, and T-PRF membrane groups at days 1, 3, and 7. (**a**) VEGF release levels in the experimental groups. (**b**) TGF-β1 release levels in the experimental groups. Groups sharing the same letter were not statistically significantly different (*p* > 0.05).

VEGF release levels were evaluated according to membrane type. No statistically significant difference was observed between the groups on day 1 (*p* > 0.05). However, statistically significant differences were detected among the groups on days 3 and 7 (*p* < 0.05). On day 3, the control group demonstrated higher VEGF release levels compared with the HA-PRF and T-PRF groups. On day 7, the highest VEGF release level was observed in the L-PRF group (Figure 4a).

Evaluation of TGF-β1 release demonstrated statistically significant differences between the groups on days 1, 3, and 7 (*p* < 0.05). In particular, the L-PRF and HA-PRF groups showed higher TGF-β1 release levels than the control and T-PRF groups on days 1 and 3 (Figure 4b).

### 2.5. Evaluation of IL-1β, IL-6, and TNF-α Gene Expression Levels

**Figure 5:**
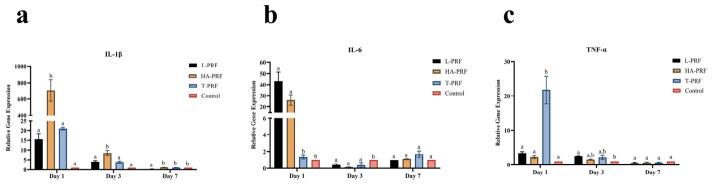
qRT-PCR analysis of inflammatory cytokine gene expression in the control, L-PRF, HA-PRF, and T-PRF membrane groups at days 1, 3, and 7. (**a**) Relative IL-1β gene expression levels. (**b**) Relative IL-6 gene expression levels. (**c**) Relative TNF-α gene expression levels. Groups sharing the same letter were not statistically significantly different (*p* > 0.05).

IL-1β gene expression levels were evaluated according to membrane type. On days 1 and 3, the HA-PRF group demonstrated significantly higher IL-1β gene expression levels compared with the other groups (*p* < 0.05). On day 7, the lowest IL-1β gene expression level was observed in the L-PRF group (Figure 5a).

Evaluation of IL-6 gene expression demonstrated that the L-PRF and HA-PRF groups showed higher IL-6 expression levels than the remaining groups on day 1 (*p* < 0.05). In contrast, the control group demonstrated significantly higher IL-6 gene expression levels than the L-PRF, HA-PRF, and T-PRF groups on day 3 (Figure 5b).

TNF-α gene expression analysis revealed that the T-PRF group demonstrated significantly higher TNF-α expression levels compared with the other groups on day 1 (*p* < 0.05). On day 3, TNF-α gene expression was significantly higher in the L-PRF group than in the control group (Figure 5c).

A comparative summary of the principal biologic findings observed in the L-PRF, HA-PRF, and T-PRF groups is presented in Table 1.

## 3. Discussion

The present study evaluated the biologic behavior of PRF membranes prepared using different tube systems on gingiva-derived mesenchymal stem cells (GMSCs). Since tissue regeneration in oral tissues begins immediately after clot formation, biomaterials capable of supporting cellular survival and regulating inflammatory events may positively influence tissue regeneration [22]. PRF has gained considerable attention in this context because it functions not only as a fibrin scaffold but also as a biologically active matrix containing platelets, leukocytes, cytokines, and growth factors involved in tissue repair [23]. In addition to supporting cellular adhesion and migration, PRF may also contribute to regulation of the local biologic microenvironment. GMSCs were selected in the present study because gingival tissues play an important role in oral tissue homeostasis and regeneration and represent an accessible mesenchymal stem cell source with regenerative and immunomodulatory properties [24]. Although PRF has been extensively investigated, studies directly comparing the effects of PRF membranes obtained from different tube materials on GMSCs remain limited. Therefore, the present study focused on cellular viability, cytoprotective, and inflammatory responses induced by L-PRF, HA-PRF, and T-PRF membranes under in vitro conditions.

The isolated cells demonstrated the characteristic properties expected for mesenchymal stem cells. Positive expression of CD105, CD90, CD73, CD44, and CD29 together with negative expression of hematopoietic and immunologic markers confirmed the mesenchymal phenotype of the cultured cells. Furthermore, successful adipogenic, osteogenic, and chondrogenic differentiation supported the stem cell characteristics of the isolated gingival cell population. These findings are consistent with the minimal criteria established by the International Society for Cellular Therapy [25].

To reduce biologic variability, both PRF membranes and GMSCs were obtained from the same donor. This approach provided a more controlled evaluation of cellular responses associated with different PRF preparation systems. Previous studies reported that PRF membranes may preserve structural integrity for several days while gradually releasing biologically active mediators over time [26,27]. In addition, release kinetics of different growth factors may vary according to fibrin organization and membrane structure, with the highest release generally occurring during the early incubation periods [28,29]. Therefore, investigation of early and intermediate incubation periods may provide valuable information regarding the temporal biologic effects of PRF membranes on cellular responses and growth factor release.

Because the MTT assay reflects mitochondrial metabolic activity of viable cells, it was used to evaluate GMSC viability and metabolic activity in the present study [30]. Ehrenfest et al. [31] previously demonstrated that L-PRF increased the viability and proliferation of bone marrow-derived mesenchymal stem cells. Similarly, Li et al. [32] reported that L-PRF promoted proliferation of stem cells derived from the apical papilla. The findings of the present study are generally consistent with these previous reports. Baygın et al. [33] also demonstrated that both L-PRF and T-PRF enhanced osteoblast viability, while T-PRF produced a more sustained proliferative response. The differences between the present findings and previous studies may be associated with variations in experimental methodology and cell type. Nevertheless, studies evaluating the proliferative and cytoprotective effects of PRF membranes prepared using different tube systems on GMSCs remain limited. The findings suggest that the biologic effects of PRF membranes on GMSC viability and metabolic activity may vary depending on the PRF preparation protocol and tube material.

Inflammatory mediators may strongly influence mesenchymal stem cell survival and tissue repair dynamics. Wedzinska et al. [34] reported that elevated IL-1β and TNF-α levels may negatively affect mesenchymal stem cell viability under certain conditions. Interestingly, despite transient increases in inflammatory mediator expression, PRF-treated groups in the present study exhibited lower cell death rates during the first incubation periods compared with controls. This observation suggests that the biologically active environment created by PRF membranes may provide protective support during early cellular adaptation.

One possible explanation for this cytoprotective effect involves the release of growth factors such as TGF-β1. PRF membranes contain platelet-derived mediators capable of regulating cellular proliferation, migration, and apoptosis [26]. In the present study, TGF-β1 release levels were generally higher in PRF-treated groups than in controls during the early incubation periods. Previous reports also indicated that TGF-β1 may participate in anti-apoptotic signaling pathways and regulation of cellular stress responses [26,35]. Therefore, the reduced cell death observed in PRF-treated groups may be associated with the biologic microenvironment generated by sustained growth factor release from the fibrin matrix.

Growth factor release from PRF membranes is closely related to fibrin architecture and membrane preparation procedures. The fibrin network may function as a biologic reservoir capable of gradually releasing platelet- and leukocyte-derived mediators over time [26]. However, previous investigations suggested that mechanical compression during membrane preparation may alter fibrin density and affect retention of growth factors within the matrix [11]. Such alterations may influence early and late release profiles and contribute to differences observed between membrane types.

This mechanism may partly explain the higher VEGF release detected in the control group on day 3. Compression of PRF membranes during preparation may have increased retention of VEGF within the fibrin matrix and delayed its release into the culture medium. In contrast, the absence of a fibrin scaffold in the control condition may have allowed faster release into the surrounding environment. Similar observations were previously reported in studies describing controlled and sustained growth factor release from PRF membranes [26].

Growth factor liberation from biomaterials may occur through immediate release, release during matrix degradation, or slow sustained diffusion [36]. Earlier studies demonstrated that T-PRF membranes possess a denser fibrin structure and may degrade more slowly than conventional L-PRF membranes [9,14]. Consequently, membranes with different types of fibrin organization may demonstrate distinct release kinetics. This mechanism may partly explain the variations observed in VEGF and TGF-β1 release profiles among PRF groups and may also contribute to the relatively lower GMSC viability observed in the T-PRF group during later incubation periods.

An interesting finding of the present study was the relatively lower cell viability observed in the T-PRF group on day 7 compared with that in the HA-PRF and L-PRF groups. Although T-PRF has been reported to form a denser and more organized fibrin network than conventional PRF preparations [9,14], this structural characteristic may not always be advantageous under in vitro conditions. A denser fibrin architecture may delay the release of growth factors and reduce the early bioavailability of biologically active mediators required for cellular metabolism and adaptation. Previous studies have demonstrated that fibrin architecture plays an important role in regulating the release kinetics of growth factors from PRF membranes [26]. Therefore, the slower release kinetics associated with T-PRF may partly explain the reduced metabolic activity observed in GMSCs compared with HA-PRF and L-PRF membranes.

Inflammatory responses are regulated through a complex network of cytokines and signaling pathways. Cytokines such as IL-1β, IL-6, and TNF-α play important roles in cellular communication, inflammatory regulation, and tissue homeostasis [37,38,39]. Because PRF membranes contain leukocytes and platelet-derived mediators, they may influence these inflammatory pathways and modulate cellular responses [40]. Ziskoven et al. [41] reported that PRF application increased TNF-α expression in periodontal ligament cells, suggesting that PRF-derived bioactive components may participate in the regulation of early inflammatory processes.

Similarly, the present study demonstrated increased proinflammatory cytokine expression during the first incubation periods, followed by a marked reduction by day 7. Rather than indicating a harmful effect, this transient increase may reflect a physiologic inflammatory response during the early stages of cellular adaptation. The subsequent reduction in cytokine expression observed at later periods suggests that PRF membranes may also contribute to regulation and resolution of inflammatory activity. Studies investigating the effects of PRF on inflammatory gene expression in GMSCs remain limited; therefore, the present findings may provide additional insight into the immunomodulatory behavior of PRF membranes prepared using different tube systems.

Mesenchymal stem cells contribute to tissue regeneration not only through differentiation but also through secretion of biologically active mediators involved in tissue homeostasis and cellular communication [42]. Previous studies demonstrated that IL-1β may modulate mesenchymal stem cell behavior through chemotactic and inflammatory signaling pathways [43,44]. In the present study, HA-PRF membranes demonstrated increased IL-1β expression during the early incubation periods. This finding may indicate that nano-hydroxyapatite-coated surfaces are capable of modulating cellular signaling and inflammatory mediator production.

The favorable biologic behavior observed in the HA-PRF group may not be explained solely by the well-known osteoconductive properties of hydroxyapatite. Previous studies have demonstrated that nano-hydroxyapatite surfaces may influence protein adsorption, cellular adhesion, and cell–material interactions through modifications in surface topography and physicochemical characteristics, thereby affecting cellular behavior independently of osteogenic differentiation [45,46]. In addition, nano-hydroxyapatite has been reported to modulate intracellular signaling pathways involved in cell survival, proliferation, and biologic activity [46]. In the present study, the increased viability and growth factor release observed in the HA-PRF group may reflect these broader biologic interactions between nano-hydroxyapatite-coated surfaces and GMSCs.

When the findings are considered collectively, PRF membranes prepared using different tube systems do not appear to behave identically at the cellular level. Variations in fibrin architecture and surface characteristics may influence growth factor release patterns, inflammatory responses, and cellular viability. Among the evaluated groups, HA-PRF and L-PRF generally demonstrated more favorable biologic behavior, whereas the denser fibrin organization associated with T-PRF may have altered release kinetics and cellular responses.

From a clinical perspective, the findings suggest that different PRF preparation systems may not be biologically interchangeable. HA-PRF and L-PRF demonstrated more favorable effects on GMSC viability and growth factor release, whereas T-PRF exhibited a distinct biologic profile that may be related to its fibrin architecture and release kinetics. These differences may become relevant when selecting PRF systems for specific regenerative applications; however, further in vivo and clinical studies are required before definitive clinical recommendations can be made.

Although the present findings suggest that HA-PRF may represent a biologically compatible alternative to conventional PRF systems, several practical considerations should be addressed before clinical implementation. Standardization and reproducibility of the nano-hydroxyapatite coating process are essential to ensure consistent biologic performance between batches. In addition, potential regulatory requirements regarding manufacturing quality control, surface characterization, and biocompatibility testing should be considered before routine clinical use. Furthermore, although nano-hydroxyapatite-coated tubes may offer a potentially cost-effective alternative to titanium tubes, additional studies evaluating production feasibility, long-term stability, and clinical safety are required before widespread clinical adoption can be recommended.

Several limitations of the present study should also be acknowledged. First, the biologic effects of L-PRF, HA-PRF, and T-PRF membranes were evaluated only under in vitro conditions, and the findings may not completely reflect the complexity of clinical tissue regeneration processes. In addition, all biologic materials were obtained from a single donor, which limited evaluation of interindividual variability. In addition, a formal sample size calculation and statistical power analysis were not performed because the present investigation was designed as an exploratory in vitro study. Relatively short incubation periods and investigation of selected biologic parameters also represent limitations of the study. Future investigations involving multiple donors, longer observation periods, and in vivo experimental models may provide more comprehensive information regarding the biologic behavior and clinical potential of nano-hydroxyapatite-based PRF systems. Furthermore, fibrin architecture, growth factor retention, and release kinetics were not directly quantified in the present study. Therefore, the mechanistic explanations proposed for the observed biologic responses should be considered hypothetical and require confirmation in future investigations specifically designed to evaluate these parameters.

## 4. Materials and Methods

### 4.1. Isolation and Characterization of Gingiva-Derived Mesenchymal Stem Cells (GMSCs)

#### 4.1.1. Isolation of GMSCs

This study was conducted in accordance with the principles of the Declaration of Helsinki and approved by the Clinical Research Ethics Committee of Eskişehir Osmangazi University (Decision No: 46; Date: 21 September 2023). The study was supported by the Eskişehir Osmangazi University Scientific Research Projects Commission (Project No: TDH-2024-3041) and the Health Institutes of Türkiye (TÜSEB) (Project No: 34399). Gingival tissue and venous blood samples were obtained from a systemically healthy nonsmoking male volunteer with no history of medication use. All procedures were performed under sterile conditions.

For isolation of GMSCs, gingival tissue samples were obtained from healthy gingival tissue during surgical extraction of impacted third molars under local anesthesia and sterile surgical conditions. Immediately after harvesting, the tissue samples were transferred into an antibiotic-containing transport medium to preserve cell viability and minimize microbial contamination.

The gingival tissues were washed three times with Dulbecco’s phosphate-buffered saline (DPBS; Capricorn Scientific, Ebsdorfergrund, Germany) to remove erythrocytes and possible microbial contaminants. Subsequently, the tissues were enzymatically digested using 2 mg/mL collagenase type II. To neutralize the enzyme activity, Dulbecco’s modified Eagle’s medium/F12 (DMEM/F12; Capricorn Scientific, Ebsdorfergrund, Germany) supplemented with 10% fetal bovine serum (FBS; Biological Industries, Beit HaEmek, Israel), 1% penicillin/streptomycin, and 1% stable glutamine was used.

The isolated cells were cultured in 6-well culture plates and incubated at 37 °C in a humidified atmosphere containing 5% CO_2_ and 95% relative humidity using a CO_2_ incubator (MCO-170M-PE; Panasonic, Kadoma, Japan). The culture medium was renewed every 3–4 days [18,19,21].

#### 4.1.2. Characterization of GMSCs

GMSCs were characterized as previously described [20,47]. Briefly, immunophenotypic characterization of GMSCs was performed using antibodies against CD90, CD73, CD44, CD29, CD105, MHC class I, CD45, CD34, CD14, CD25, and MHC class II (BioLegend, San Diego, CA, USA). Flow cytometric analysis and differentiation assays were performed to confirm the mesenchymal stem cell phenotype of the isolated cells. Flow cytometry was performed using a NovoCyte D3005 flow cytometry (Agilent, Santa Clara, CA, USA). The data were analyzed using NovoExpress flowncytometry software (version 1.5.0, Agilent).

In addition, the isolated cells were differentiated into adipogenic, osteogenic, and chondrogenic lineages. The adipogenic medium, MEM (Invitrogen/GIBCO, Waltham, MA, USA), was supplemented with 10% FBS (Invitrogen/GIBCO, Waltham, MA, USA), 0.5 mM isobutylmethylxanthine (IBMX; Sigma-Aldrich, St. Louis, MO, USA), 10^−6^ M dexamethasone (Sigma-Aldrich, St. Louis, MO, USA), 10 μg/mL insulin (Invitrogen/GIBCO, Waltham, MA, USA), 200 μM indomethacin (Sigma-Aldrich, St. Louis, MO, USA), and 0.2% Primocin (InvivoGen, San Diego, CA, USA) for 4 weeks. The medium was replaced twice weekly. Adipogenic differentiation was confirmed by histochemical staining of intracellular lipid droplets using Oil Red O (Sigma-Aldrich, St. Louis, MO, USA).

For osteogenic differentiation, the cells were cultured in differentiation medium consisting of MEM (Invitrogen/GIBCO, Waltham, MA, USA) supplemented with 100 nM dexamethasone (Sigma-Aldrich, St. Louis, MO, USA), 0.05 μM ascorbate-2-phosphate (Wako Chemicals, Richmond, VA, USA), 10 mM β-glycerophosphate (Sigma-Aldrich, St. Louis, MO, USA), 0.2% Primocin (InvivoGen, San Diego, CA, USA), and 10% FBS (Invitrogen/GIBCO, MA, USA). The medium was replaced twice weekly. At the end of the fourth week, osteogenic differentiation was confirmed by histochemical staining of mineralized nodules using Alizarin Red S (Sigma-Aldrich, St. Louis, MO, USA).

For chondrogenic differentiation, 2.5 × 10^5^ cells were transferred into a 15 mL polypropylene tube and centrifuged at 1300× *g* for 5 min to form a three-dimensional pellet micromass. The micromasses were cultured in chondrogenic medium consisting of high-glucose DMEM (GIBCO) supplemented with 10 ng/mL transforming growth factor-β1 (TGF-β1; PHG0021 MyBiosource, San Diego, CA, USA), 50 μg/mL ascorbate-2-phosphate (Wako Chemicals, Richmond, VA, USA), 0.1 μM dexamethasone (Sigma-Aldrich, St. Louis, MO, USA), 100 μg/mL sodium pyruvate (Sigma-Aldrich, St. Louis, MO, USA), 40 μg/mL proline (Merck, Darmstadt, Germany), 50 mg/mL ITS premix (BD Biosciences, San Jose, CA, USA), and 0.2% Primocin (InvivoGen, CA, USA). The medium was changed twice weekly for 2 weeks. Chondrogenic differentiation was confirmed by Alcian Blue and Safranin O (Sigma-Aldrich, St. Louis, MO, USA) staining after fixation in 4% paraformaldehyde and paraffin embedding.

### 4.2. Production and Analysis of Nano-Hydroxyapatite-Coated Tubes

Nano-hydroxyapatite-coated tubes were produced under laboratory conditions as part of the present study. Glass tubes [11] (Borosil, Mumbai, India) and titanium tubes [9] (Firdevs Dental Medikal Limited Şirketi, Konya, Türkiye) were commercially obtained.

An ultrasonic spray pyrolysis (USP) system was used for nano-hydroxyapatite (nHA) coating. Prior to coating, the glass tubes were cleaned with ethyl alcohol and exposed to hot air flow for 4 min to prepare the surfaces for coating.

For preparation of the coating solution, calcium nitrate tetrahydrate [Ca(NO_3_)_2_·4H_2_O] (AFG BioScience, Northbrook, IL, USA; 98% purity) and ammonium dihydrogen phosphate [NH_4_H_2_PO_4_] (Sigma-Aldrich, St. Louis, MO, USA) were used as calcium and phosphorus precursors, respectively. The coating solution was prepared at a concentration of 0.5 M according to the previously described protocol [48].

The coating procedure was performed in the USP system by inserting and withdrawing the nozzle coaxially into the tube lumen. This protocol was designed to obtain a homogeneous longitudinal nano-hydroxyapatite film on the inner surface of the tubes. During each cycle, the nozzle was inserted to a predetermined depth, followed by brief spraying and controlled withdrawal. The coating procedure lasted approximately 5–10 min, and different cycle numbers were evaluated.

**Figure 6:**
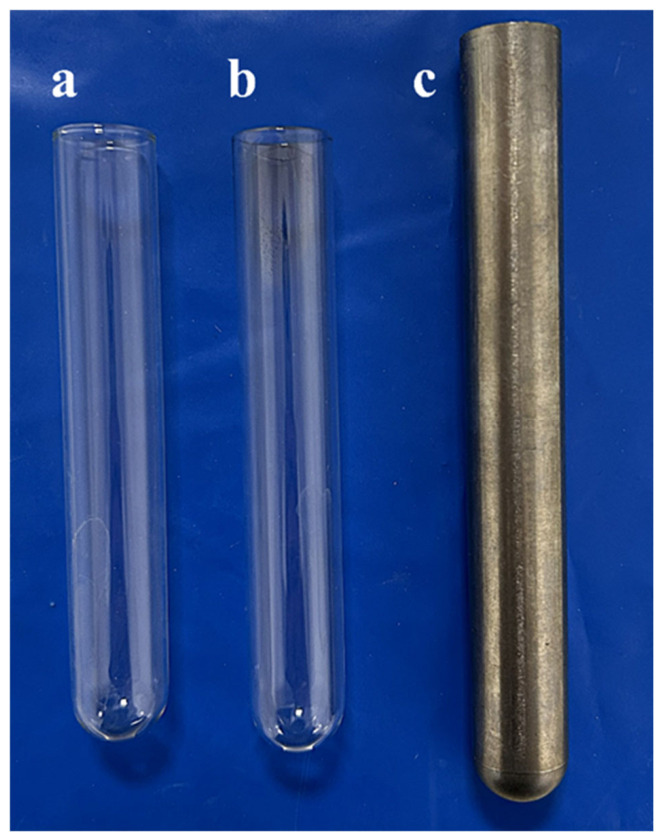
Representative images of the tube systems used for PRF preparation: (**a**) conventional glass tube, (**b**) nano-hydroxyapatite-coated tube, and (**c**) titanium tube.

During the coating procedure, the substrate temperature was maintained at 300–350 °C. The nozzle movement and spraying steps were synchronized using a programmable logic controller (PLC) system. The most homogeneous coating integrity and film structure were achieved using a 0.5 M solution at 300–350 °C [48]. Representative images of the tube systems used in the study are presented in Figure 6.

### 4.3. Preparation of PRF Membranes

Venous blood samples were collected from the same donor under sterile conditions for preparation of PRF membranes. A total of 120 mL venous blood was collected and immediately transferred into four conventional glass tubes, four nano-hydroxyapatite-coated glass tubes, and four titanium tubes without anticoagulants, with each tube containing 10 mL blood.

For preparation of leukocyte platelet-rich fibrin (L-PRF), hydroxyapatite platelet-rich fibrin (HA-PRF), and titanium-prepared platelet-rich fibrin (T-PRF) membranes, blood samples collected in conventional glass tubes, nano-hydroxyapatite-coated tubes, and titanium tubes were centrifuged at 2700 rpm for 12 min using a tabletop centrifuge device (Elektro-Mag, Istanbul, Türkiye). HA-PRF membranes were obtained using the nano-hydroxyapatite-coated tubes developed in the present study. To minimize potential confounding effects related to centrifugation parameters, all PRF groups were prepared using the same centrifugation protocol. Therefore, the primary variable investigated in this study was tube material and surface characteristics.

**Figure 7:**
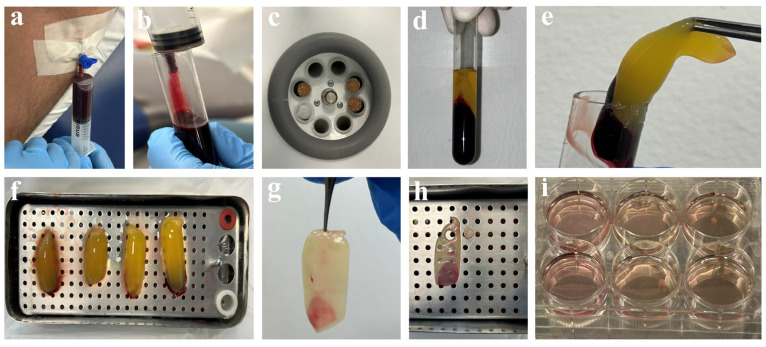
(**a**) Blood collection from a volunteer individual, (**b**) Transfer of blood into tubes, (**c**) Placement of tubes into the centrifuge device, (**d**) Obtaining the PRF clot after centrifugation, (**e**) Retrieval of the PRF clot using a pressure device, (**f**) Placement of PRF clots into metal boxes, (**g**) PRF membrane obtained as a result of metal lid compression, (**h**) Conversion of the PRF membrane into 4 mm discs using a biopsy punch, (**i**) Random placement of the disc-shaped membranes into 6-well plates.

Following centrifugation, PRF clots located between the red blood cell layer and acellular plasma layer were carefully removed from the tubes using sterile forceps. The erythrocyte-rich lower portions of the clots were carefully trimmed using sterile scissors. Subsequently, the PRF clots were gently compressed using a standardized metal PRF box to obtain PRF membranes [10]. PRF membranes were cut into standardized 4 mm discs using a biopsy punch (Plasti-Med, Istanbul, Türkiye) (Figure 7).

One PRF membrane disc was randomly placed at the center of each well using sterile forceps. Control wells without PRF membranes were prepared for each incubation period, and culture medium supplemented with 10% FBS (Biological Industries, Beit HaEmek, Israel) was used as the control condition. In the 7-day incubation group, the culture medium was renewed on day 3.

**Figure 8:**
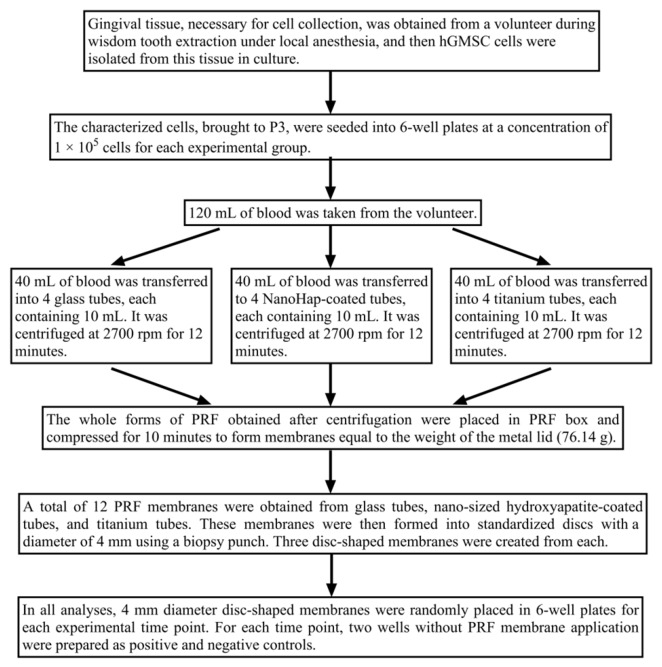
Schematic illustration of the experimental design and preparation workflow of PRF membranes used in the study.

All PRF membranes were prepared by the same investigator under standardized laboratory conditions immediately after blood collection to minimize procedural variability. The experimental design and PRF preparation workflow are summarized in Figure 8.

### 4.4. In Vitro Cell Viability and Metabolic Activity Analysis (MTT Assay)

Cell viability and metabolic activity of GMSCs cultured with different PRF membranes were evaluated using the MTT assay. Briefly, the PRFs were first positioned into empty 24-well plates, and the cells were subsequently seeded directly onto the PRFs. On days 1, 3, and 7, the cells in each well were detached using trypsin-EDTA (Capricorn Scientific, Ebsdorfergrund, Germany), re-seeded into 96-well culture plates at equal densities, and allowed to adhere. Following adherence, the MTT solution was added to each well and incubated at 37 °C for 4 h. After incubation, the supernatants were carefully removed, and dimethyl sulfoxide (DMSO) was added to dissolve the formazan crystals. Blank wells containing cell-free culture medium were utilized for background correction. The optical density (OD) values were measured at 570 nm using a microplate reader (BIOTEK ELx808IU, BioTek, Winooski, VT, USA). The data were analyzed using Gen5 software (version 3.02).

Relative cell viability and metabolic activity were calculated based on OD measurements and compared among the study groups [49]. All experimental conditions were performed in triplicate.

### 4.5. Evaluation of Cell Death by Annexin V/PI Assay

For apoptosis analysis, GMSCs were cultured directly on the PRF membranes placed in 24-well plates. On days 1, 3, and 7, cells were detached from the PRF surfaces using trypsin-EDTA and collected for analysis.

Cell death analysis was performed using an Annexin V/propidium iodide (PI) flow cytometry assay (Elabscience, Houston, TX, USA). The harvested cells were washed with phosphate-buffered saline (PBS, Capricorn Scientific, Ebsdorfergrund, Germany) and stained with Annexin V and PI solution according to the manufacturer’s instructions.

Flow cytometric analysis was performed using a NovoCyte 3500 flow cytometer (Agilent Technologies, CA, USA). A minimum of 10,000 events were acquired for each sample. Percentages of viable, early apoptotic, late apoptotic, and necrotic cells were calculated separately for each study group. Flow cytometric data were analyzed using NovoExpress software (version 1.5.0, Agilent Technologies, CA, USA) [50]. 

### 4.6. VEGF and TGF-β1 Release Profiles

GMSCs were cultured directly on PRF membranes placed in 24-well plates. On days 1, 3, and 7, culture supernatants were collected and stored at −80 °C until analysis.

VEGF and TGF-β1 release levels were evaluated using an enzyme-linked immunosorbent assay (ELISA). Commercial ELISA kits specific for VEGF and TGF-β1 (Elabscience, Houston, TX, USA) were used according to the manufacturer’s instructions. All measurements were performed in triplicate. Absorbance measurements were obtained using a microplate reader (BIOTEK ELx808IU, BioTek, VT, USA), and growth factor concentrations were calculated using standard calibration curves [51]. The data were analyzed using Gen5 software (version 3.02).

### 4.7. Evaluation of IL-1β, IL-6, and TNF-α Gene Expression

GMSCs were cultured directly on PRF membranes placed in 24-well plates. On days 1, 3, and 7, cells were harvested, and total RNA was isolated for gene expression analysis.

Total RNA was isolated from GMSCs cultured with PRF membranes using the A.B.T RNA Purification Kit (Atlas Biyoteknoloji, Ankara, Türkiye) according to the manufacturer’s instructions. RNA concentration and purity were determined spectrophotometrically using a NanoDrop-One spectrophotometer (Thermo Fisher Scientific, Waltham, MA, USA), and only RNA samples with an A260/A280 ratio between 1.8 and 2.0 were used. Complementary DNA (cDNA) synthesis was performed using 1 μg of total RNA and a cDNA Synthesis Kit with RNase Inhibitor (Atlas Biyoteknoloji, Ankara, Türkiye) according to the manufacturer’s protocol.

Quantitative real-time polymerase chain reaction (qRT-PCR) was performed to evaluate IL-1β, IL-6, and TNF-α gene expression using 2× qPCR SYBR Green Master Mix (Atlas Biyoteknoloji, Ankara, Türkiye) on a Rotor-Gene Q5 Plex + HRM Real-Time PCR System (Qiagen, Hilden, Germany). The thermal cycling conditions consisted of an initial denaturation at 95 °C for 5 min, followed by 40 cycles of denaturation at 95 °C for 30 s and annealing/extension at 59 °C for 60 s. All qRT-PCR reactions were performed in triplicate. Relative gene expression levels were calculated using the 2^−ΔΔCt method and normalized against the housekeeping gene GAPDH [49].

Primer sequences used for qRT-PCR analysis are presented in Table 2.

### 4.8. Statistical Analysis

Statistical analyses were performed using IBM SPSS Statistics software (IBM SPSS statistics version 22.0 IBM Corp., Armonk, NY, USA). Normality of data distribution was evaluated using the Shapiro–Wilk test. For MTT, ELISA, and qRT-PCR analyses, two-way analysis of variance (ANOVA) was used to evaluate the effects of PRF group (L-PRF, HA-PRF, and T-PRF), incubation period (days 1, 3, and 7), and their interaction. Post hoc multiple comparisons were performed using Tukey’s test. Data are expressed as mean ± standard deviation (SD). All experiments were performed in triplicate, and a *p* value < 0.05 was considered statistically significant. All experiments were performed in triplicate as technical replicates for each experimental condition and time point.

## 5. Conclusions

Within the limitations of this in vitro study, HA-PRF membranes showed no evidence of cytotoxicity and demonstrated biologic responses comparable to those observed with conventional L-PRF. HA-PRF and L-PRF exhibited comparable effects on cell viability, metabolic activity, growth factor release, and inflammatory cytokine expression, whereas T-PRF demonstrated relatively less favorable cellular responses during the later incubation periods.

Overall, the findings suggest that HA-PRF may represent a biologically compatible alternative to conventional L-PRF for PRF preparation. Although the present findings were obtained under in vitro conditions, further experimental and clinical studies are required to better clarify the long-term biologic behavior and regenerative potential of HA-PRF membranes.

## Figures and Tables

**Table 1:** Summary of the principal biologic findings observed in L-PRF, HA-PRF, and T-PRF groups.

Biological Parameter	L-PRF	HA-PRF	T-PRF
Cell viability and metabolic activity	Favorable response; highest values during early incubation periods	Favorable response; comparable to L-PRF on day 7	Relatively lower metabolic activity on day 7
Cytotoxicity (Annexin V/PI)	No evidence of cytotoxicity	No evidence of cytotoxicity	No evidence of cytotoxicity
VEGF release	Highest VEGF release on day 7	Moderate VEGF release	Lower VEGF release profile
TGF-β1 release	Increased release during early periods	Increased release during early periods	Lower release tendency compared with L-PRF and HA-PRF
IL-1β expression	Transient increase followed by reduction	Highest early expression followed by reduction	Transient increase followed by reduction
IL-6 expression	Time-dependent changes	Time-dependent changes	Time-dependent changes
TNF-α expression	Increased expression on day 3	Time-dependent changes	Highest expression on day 1
Overall biologic response	Favorable	Favorable	Relatively less favorable during later incubation periods

Abbreviations: L-PRF, leukocyte platelet-rich fibrin; HA-PRF, nano-hydroxyapatite platelet-rich fibrin; T-PRF, titanium platelet-rich fibrin. The table summarizes the principal biologic findings and should be interpreted together with the detailed results and statistical analyses presented in the manuscript.

**Table 2:** Primer sequences used for qRT-PCR analysis.

Target Gene	Forward Primer (5′–3′)	Reverse Primer (3′–5′)
TNF-α	CATGATCCGGGACGTGGAG	TTCGAGAAGATGATCTGACTGCC
IL-6	CCCACCGGGAACGAAAGAG	GGACCGAAGGCGCTTGT
IL-1β	GCCAATCTTCATTGCTCAAGTGT	AGCCATCATTTCACTGGCGA

## Data Availability

The datasets generated and/or analyzed during the current study are available from the corresponding author on reasonable request.

## Abbreviations

The following abbreviations are used in this manuscript:

DPBS	Dulbecco’s phosphate-buffered saline
DMEM/F12	Dulbecco’s modified Eagle’s medium/F12
ELISA	Enzyme-linked immunosorbent assay
FBS	Fetal bovine serum
GMSCs	Gingiva-derived mesenchymal stem cells
HA-PRF	Hydroxyapatite platelet-rich fibrin
IL	Interleukin
L-PRF	Leukocyte platelet-rich fibrin
MTT	3-(4,5-dimethylthiazol-2-yl)-2,5-diphenyltetrazolium bromide
PBS	Phosphate-buffered saline
PLC	Programmable logic controller
PRF	Platelet-rich fibrin
qRT-PCR	Quantitative real-time polymerase chain reaction
SEM	Scanning electron microscopy
TGF-β1	Transforming growth factor beta-1
TNF-α	Tumor necrosis factor alpha
T-PRF	Titanium-prepared platelet-rich fibrin
USP	Ultrasonic spray pyrolysis
VEGF	Vascular endothelial growth factor

## References

[1] Miron R.J., Fujioka-Kobayashi M., Bishara M., Zhang Y., Hernandez M., Choukroun J. (2016). Platelet-Rich Fibrin and Soft Tissue Wound Healing: A Systematic Review. Tissue Eng. Part B Rev..

[2] Dohan D.M., Choukroun J., Diss A., Dohan S.L., Dohan A.J., Mouhyi J., Gogly B. (2006). Platelet-rich fibrin (PRF): A second-generation platelet concentrate. Part II: Platelet-related biologic features. Oral Surg. Oral Med. Oral Pathol. Oral Radiol. Endodontol..

[3] Tsujino T., Takahashi A., Yamaguchi S., Watanabe T., Isobe K., Kitamura Y., Tanaka T., Nakata K., Kawase T. (2019). Evidence for Contamination of Silica Microparticles in Advanced Platelet-Rich Fibrin Matrices Prepared Using Silica-Coated Plastic Tubes. Biomedicines.

[4] Yamaguchi S., Aizawa H., Sato A., Tsujino T., Isobe K., Kitamura Y., Watanabe T., Okudera H., Mourão C.F., Kawase T. (2020). Concentrated Growth Factor Matrices Prepared Using Silica-Coated Plastic Tubes Are Distinguishable From Those Prepared Using Glass Tubes in Platelet Distribution: Application of a Novel Near-Infrared Imaging-Based, Quantitative Technique. Front. Bioeng. Biotechnol..

[5] Miron R.J., Kawase T., Dham A., Zhang Y., Fujioka-Kobayashi M., Sculean A. (2021). A technical note on contamination from PRF tubes containing silica and silicone. BMC Oral Health.

[6] Thon J.N., Italiano J.E. (2012). Platelets: Production, morphology and ultrastructure. Handb. Exp. Pharmacol..

[7] Bowen R.A., Remaley A.T. (2014). Interferences from blood collection tube components on clinical chemistry assays. Biochem. Med..

[8] Mourão C.F., Pinto N., Panos I.A., Juliasse L., Gurgel B.V. (2024). RE: Fibrin Network and Platelets Densities in Platelet-Rich Fibrin (PRF) Membranes Produced from Plastic Tubes Without Additives—A New Approach to PRF Clinical Use. J. Maxillofac. Oral Surg..

[9] Tunalı M., Özdemir H., Küçükodacı Z., Akman S., Yaprak E., Toker H., Fıratlı E. (2014). A Novel Platelet Concentrate: Titanium-Prepared Platelet-Rich Fibrin. BioMed Res. Int..

[10] Ravi S., Santhanakrishnan M. (2020). Mechanical, chemical, structural analysis and comparative release of PDGF-AA from L-PRF, A-PRF and T-PRF—An in vitro study. Biomater. Res..

[11] Dohan Ehrenfest D.M., Pinto N.R., Pereda A., Jiménez P., Del Corso M., Kang B.-S., Nally M., Lanata N., Wang H.-L., Quirynen M. (2018). The impact of the centrifuge characteristics and centrifugation protocols on the cells, growth factors, and fibrin architecture of a leukocyte- and platelet-rich fibrin (L-PRF) clot and membrane. Platelets.

[12] Tsujino T., Masuki H., Nakamura M., Isobe K., Kawabata H., Aizawa H., Watanabe T., Kitamura Y., Okudera H., Okuda K. (2019). Striking Differences in Platelet Distribution between Advanced-Platelet-Rich Fibrin and Concentrated Growth Factors: Effects of Silica-Containing Plastic Tubes. J. Funct. Biomater..

[13] Masuki H., Isobe K., Kawabata H., Tsujino T., Yamaguchi S., Watanabe T., Sato A., Aizawa H., Mourão C.F., Kawase T. (2020). Acute cytotoxic effects of silica microparticles used for coating of plastic blood-collection tubes on human periosteal cells. Odontology.

[14] Tunalı M., Ercan E., Pat S., Sarıca E., Bağla A.G., Aytürk N., Sıddıkoğlu D., Bilgin V. (2024). Nano-titanium coating on glass surface to improve platelet-rich fibrin (PRF) quality. J. Mater. Sci. Mater. Med..

[15] Wang J., Sun Y., Liu Y., Yu J., Sun X., Wang L., Zhou Y. (2022). Effects of platelet-rich fibrin on osteogenic differentiation of Schneiderian membrane derived mesenchymal stem cells and bone formation in maxillary sinus. Cell Commun. Signal..

[16] Iozon S., Caracostea G.V., Páll E., Şoriţău O., Mănăloiu I.D., Bulboacă A.-E., Lupşe M., Mihu C.M., Roman A.L. (2020). Injectable platelet-rich fibrin influences the behavior of gingival mesenchymal stem cells. Rom. J. Morphol. Embryol..

[17] Hong S., Chen W., Jiang B. (2018). A Comparative Evaluation of Concentrated Growth Factor and Platelet-rich Fibrin on the Proliferation, Migration, and Differentiation of Human Stem Cells of the Apical Papilla. J. Endod..

[18] Kim D., Lee A.E., Xu Q., Zhang Q., Le A.D. (2021). Gingiva-Derived Mesenchymal Stem Cells: Potential Application in Tissue Engineering and Regenerative Medicine—A Comprehensive Review. Front. Immunol..

[19] Jin S.H., Lee J.E., Yun J., Kim I., Ko Y., Park J.B. (2014). Isolation and characterization of human mesenchymal stem cells from gingival connective tissue. J. Periodontal Res..

[20] Uysal O., Erybeh H., Canbek M., Ekenel E.Q., Gunes S., Büyükköroğlu G., Sevimli T.S., Cemrek F., Sariboyaci A.E. (2024). Stem Cell-Based or Cell-Free Gene Therapy in Chondrocyte Regeneration: Synovial Fluid-Derived Mesenchymal Stem Cell Exosomes. Curr. Mol. Med..

[21] Du L., Yang P., Ge S. (2016). Isolation and characterization of human gingiva-derived mesenchymal stem cells using limiting dilution method. J. Dent. Sci..

[22] Barrientos S., Stojadinovic O., Golinko M.S., Brem H., Tomic-Canic M. (2008). PERSPECTIVE ARTICLE: Growth factors and cytokines in wound healing. Wound Repair Regen..

[23] Fujioka-Kobayashi M., Schaller B., De Almeida Barros Mourão C.F., Zhang Y., Sculean A., Miron R.J. (2021). Biological characterization of an injectable platelet-rich fibrin mixture consisting of autologous albumin gel and liquid platelet-rich fibrin (Alb-PRF). Platelets.

[24] Fawzy El-Sayed K.M., Dörfer C.E. (2016). Gingival Mesenchymal Stem/Progenitor Cells: A Unique Tissue Engineering Gem. Stem Cells Int..

[25] Dominici M., Le Blanc K., Mueller I., Slaper-Cortenbach I., Marini F.C., Krause D.S., Deans R.J., Keating A., Prockop D.J., Horwitz E.M. (2006). Minimal criteria for defining multipotent mesenchymal stromal cells. The International Society for Cellular Therapy position statement. Cytotherapy.

[26] Kobayashi E., Flückiger L., Fujioka-Kobayashi M., Sawada K., Sculean A., Schaller B., Miron R.J. (2016). Comparative release of growth factors from PRP, PRF, and advanced-PRF. Clin. Oral Investig..

[27] He L., Lin Y., Hu X., Zhang Y., Wu H. (2009). A comparative study of platelet-rich fibrin (PRF) and platelet-rich plasma (PRP) on the effect of proliferation and differentiation of rat osteoblasts in vitro. Oral Surg. Oral Med. Oral Pathol. Oral Radiol. Endodontol..

[28] Fujioka-Kobayashi M., Miron R.J., Hernandez M., Kandalam U., Zhang Y., Choukroun J. (2017). Optimized Platelet-Rich Fibrin with the Low-Speed Concept: Growth Factor Release, Biocompatibility, and Cellular Response. J. Periodontol..

[29] Ghanaati S., Booms P., Orlowska A., Kubesch A., Lorenz J., Rutkowski J., Landes C., Sader R., Kirkpatrick C., Choukroun J. (2014). Advanced Platelet-Rich Fibrin: A New Concept for Cell-Based Tissue Engineering by Means of Inflammatory Cells. J. Oral Implantol..

[30] Mosmann T. (1983). Rapid colorimetric assay for cellular growth and survival: Application to proliferation and cytotoxicity assays. J. Immunol. Methods.

[31] Ehrenfest D.M.D., Doglioli P., de Peppo G.M., Del Corso M., Charrier J.-B. (2010). Choukroun’s platelet-rich fibrin (PRF) stimulates in vitro proliferation and differentiation of human oral bone mesenchymal stem cell in a dose-dependent way. Arch. Oral Biol..

[32] Li X., Yang H., Zhang Z., Yan Z., Lv H., Zhang Y., Wu B. (2018). Platelet-rich fibrin exudate promotes the proliferation and osteogenic differentiation of human periodontal ligament cells in vitro. Mol. Med. Rep..

[33] Baygın M., Çakiris A., Tak A.Y., Abacı N., Ekmekçi S.S., Köseoğlu B.G. (2024). In vitro comparison of the effects of titanium-prepared platelet-rich fibrin and leukocyte platelet-rich fibrin on osteoblast behavior and their gen expression. BMC Oral Health.

[34] Wedzinska A., Figiel-Dabrowska A., Kozlowska H., Sarnowska A. (2021). The Effect of Proinflammatory Cytokines on the Proliferation, Migration and Secretory Activity of Mesenchymal Stem/Stromal Cells (WJ-MSCs) under 5% O_2_ and 21% O_2_ Culture Conditions. J. Clin. Med..

[35] Verboket R., Herrera-Vizcaíno C., Thorwart K., Booms P., Bellen M., Al-Maawi S., Sader R., Marzi I., Henrich D., Ghanaati S. (2018). Influence of concentration and preparation of platelet rich fibrin on human bone marrow mononuclear cells (in vitro). Platelets.

[36] Galler K.M., D’souza R.N., Hartgerink J.D., Schmalz G. (2011). Scaffolds for Dental Pulp Tissue Engineering. Adv. Dent. Res..

[37] Guo S., DiPietro L.A. (2010). Critical Review in Oral Biology & Medicine: Factors Affecting Wound Healing. J. Dent. Res..

[38] Eming S.A., Krieg T., Davidson J.M. (2007). Inflammation in Wound Repair: Molecular and Cellular Mechanisms. J. Investig. Dermatol..

[39] Landén N.X., Li D., Ståhle M. (2016). Transition from inflammation to proliferation: A critical step during wound healing. Cell. Mol. Life Sci..

[40] Miron R.J., Zucchelli G., Pikos M.A., Salama M., Lee S., Guillemette V., Fujioka-Kobayashi M., Bishara M., Zhang Y., Wang H.-L. (2017). Use of platelet-rich fibrin in regenerative dentistry: A systematic review. Clin. Oral Investig..

[41] Ziskoven C., Jäger M., Zilkens C., Bloch W., Brixius K., Krauspe R. (2010). Oxidative stress in secondary osteoarthritis: From cartilage destruction to clinical presentation?. Orthop. Rev..

[42] Caplan A.I., Dennis J.E. (2006). Mesenchymal stem cells as trophic mediators. J. Cell. Biochem..

[43] Kwon D.S., Gao X., Liu Y.B., Dulchavsky D.S., Danyluk A.L., Bansal M., Chopp M., McIntosh K., Arbab A.S., Dulchavsky S.A. (2008). Treatment with bone marrow-derived stromal cells accelerates wound healing in diabetic rats. Int. Wound J..

[44] Chen L., Tredget E.E., Wu P.Y.G., Wu Y. (2008). Paracrine Factors of Mesenchymal Stem Cells Recruit Macrophages and Endothelial Lineage Cells and Enhance Wound Healing. PLoS ONE.

[45] Webster T. (2000). Enhanced functions of osteoblasts on nanophase ceramics. Biomaterials.

[46] Mendonça G., Mendonça D.B., Aragão F.J., Cooper L.F. (2008). Advancing dental implant surface technology—From micron- to nanotopography. Biomaterials.

[47] Altug B., Soykan M.N., Eyubova S., Sariboyaci A.E., Dogan C., Ozalp O., Atalay E. (2024). Crosstalk among miR-29, α-SMA, and TGFβ1 /β3 in melatonin-induced exosome (Mel-prExo) treated human limbal mesenchymal stem cells (hLMSCs): An insight into scarless healing of the cornea. BioFactors.

[48] Jokanovic V., Uskokovic D. (2005). Calcium Hydroxyapatite Thin Films on Titanium Substrates Prepared by Ultrasonic Spray Pyrolysis. Mater. Trans..

[49] Rodríguez-Lozano F.J., López-García S., García-Bernal D., Sanz J.L., Lozano A., Pecci-Lloret M.P., Melo M., López-Ginés C., Forner L. (2021). Cytocompatibility and bioactive properties of the new dual-curing resin-modified calcium silicate-based material for vital pulp therapy. Clin. Oral Investig..

[50] Kargarpour Z., Nasirzade J., Panahipour L., Miron R.J., Gruber R. (2021). Platelet-Rich Fibrin Decreases the Inflammatory Response of Mesenchymal Cells. Int. J. Mol. Sci..

[51] Yang H., Gao L.-N., An Y., Hu C.-H., Jin F., Zhou J., Jin Y., Chen F.-M. (2013). Comparison of mesenchymal stem cells derived from gingival tissue and periodontal ligament in different incubation conditions. Biomaterials.

